# Magnetic resonance evaluation of coronary anatomy, first-pass myocardial perfusion and late gadolinium enhancement in children with acquired and congenital heart disease

**DOI:** 10.1186/1532-429X-17-S1-P144

**Published:** 2015-02-03

**Authors:** Dilachew Adebo, John Brownlee, John M Morales

**Affiliations:** Pediatric Cardiology, Driscoll Children’s Hospital, Corpus Christi, TX USA; Texas A and M University, Corpus Christi, TX USA; Cardiothoracic Surgery, Driscoll Children’s Hospital, Corpus Christi, TX USA

## Background

Cardiovascular magnetic resonance (CMR) has expanded its role in the diagnosis and management of congenital heart disease and acquired heart disease in children. However, there are limited studies evaluating the role of cardiac magnetic resonance in delineating the anatomy of coronary arteries along with assessment of first pass myocardial perfusion in children. The purpose of this study is to evaluate the extensive use of CMR for delineating coronary anatomy, evaluating first pass myocardial perfusion and late gadolinium enhancement in children with acquired and congenital heart disease.

## Methods

Retrospective review of 37 consecutive CMR Whole Heart T2 Prep coronary angiography studies of patients with congenital and acquired heart disease. The subjects had CMR from December, 2013 to September, 2014. Results of first pass myocardial perfusion study (at rest and with adenosine stress) and myocardial inversion recovery for delayed myocardial enhancement imaging were also reviewed.

## Results

**Figure 1:**
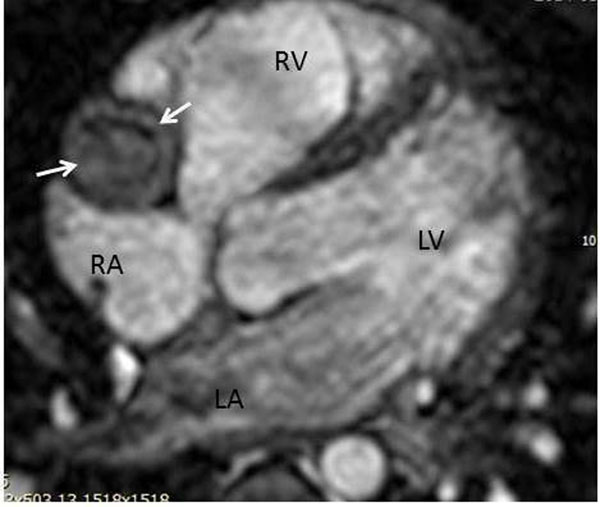
**Whole heart T2 prep coronary imaging of 5 year old patient with Kawasaki disease shows giant aneurysm of right coronary atery in right atrioventricular groove (arrows) with occlusive thrombus in the lumen.** RV: right ventricle; LV: left ventricle; RA right atrium; LA left atrium.

The median age at the time of CMR was 11 years with range of 3 months to 25 years; 26 male and 11 female subjects. The origins and proximal course of coronary were well demonstrated by CMR in 92% (34/37) of the cases. Five patients (13.5%) had multiple coronary artery aneurysms related to Kawasaki disease (1 and 2). One of these patients had giant aneurysm of right coronary artery with occlusive thrombus (Figure 1). In this patient, there was first-pass myocardial perfusion defect both at rest and with Adenosine stress consistent with fixed perfusion defect (irreversible ischemic change). This patient also had evidence of delayed myocardial enhancement involving the subendocardium which correlates with the area of first-pass myocardial perfusion defect. 16 patients (43%) had right-ventricle to pulmonary artery conduit placement and CMR delineated the coronary artery anatomy in preparartion for percutaneous pulmonary valve (Melody valve) implantation. Two patients (5%) had anomalous aortic origin of coronary arteries. Post-repair CMR showed widely patent unroofed coronary artery with normal first pass myocardial perfusion (at rest and with adenosine stress) and no evidence of delayed myocardial enhancement. Two patients (5%) were with Marfan's syndrome after valve sparing aortic root replacement with coronary reimplantation. CMR showed widely patent re-implanted coronary arteries. Four patients (11%) were after arterial switch operation for dextro-transposition of great arteries and CMR delineated their coronary anatomy with no evidence of myocardial scar or fibrosis. Two patients had unsatisfactory image quality due to dental brace and spinal fusion rod artifact. One infant (3 month old) had unsatisfactory image quality due to very fast heart rate.

**Figure 2:**
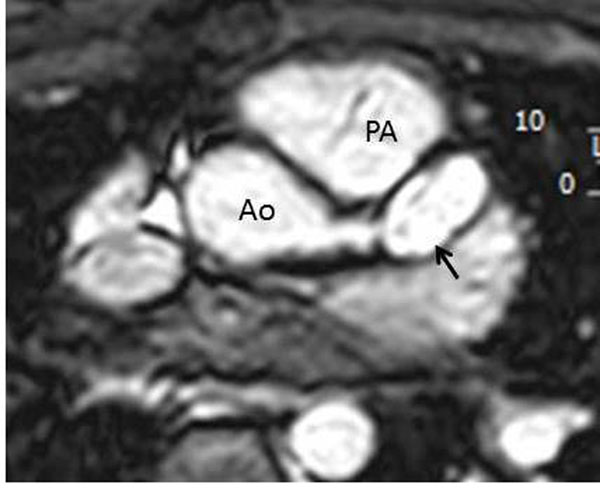
**Whole heart T2 prep coronary imaging in a 16 year old patient with history of Kawasaki disease after coronary bypass surgery.** There is stenosis of left main coronary artery with distal aneurysm (arrow) and occlusion of left decending atery.

## Conclusions

Cardiac magnetic resonance imaging can reliably evaluate the coronary anatomy, first pass myocardial perfusion defect and myocardial scar in diverse group of children with acquired and congenital heart diseases.

## Funding

None.

